# Intention to use maternal waiting home and its predictors among pregnant women in Ethiopia: systematic review and meta-analysis

**DOI:** 10.1186/s40001-023-01248-7

**Published:** 2023-08-07

**Authors:** Natnael Atnafu Gebeyehu, Yibeltal Assefa, Kelemu Abebe

**Affiliations:** 1 Department of Midwifery, College of Medicine and Health Science, Wolaita Sodo University, Sodo, Ethiopia; 2 Department of Public Health, College of Medicine and Health Science, Wolaita Sodo University, Sodo, Ethiopia

**Keywords:** Intention, Maternity waiting home, Pregnant women, Meta-analysis, Ethiopia

## Abstract

**Background:**

A high maternal death rate is a result of maternal delays in seeking emergency obstetric care, particularly in countries with limited resources like Ethiopia. Utilizing maternity waiting homes is a strategy to overcome geographical barriers and improve maternal and neonatal health outcomes. Pregnant women must intend to use this service in addition to it being available. Therefore, the goal of this study was to assess pregnant women's intentions to use maternity waiting homes and associated characteristics.

**Methods:**

PubMed, Google Scholar, Scopus, Science Direct, and online institutional repository homes were searched. Data were extracted using Microsoft Excel and analyzed using STATA statistical software (v. 14). Publication bias was checked by forest plot, Begg's, and Egger's tests. To look for heterogeneity, *I*^2^ was computed, and an overall estimated analysis was carried out. Subgroup analysis was done by study region, sample size, and publication. The pooled odds ratio for associated factors was also computed.

**Results:**

Out of 258 articles assessed, 8 studies with 4111 study participants met the criteria and were included in this study. The pooled prevalence of intention to use maternity waiting home was 52.25% (95% CI 45.88–58.66), *I*^2^ = 93.8%). Amhara region had a higher intention to use maternal waiting for home prevalence (63.5%), per subgroup analysis. In studies with sample sizes higher than 5000, the usage of maternity waiting homes was less prevalent (45.2%). Between published research (52.9%) and unpublished studies (51.3%), there was no appreciable difference in the intention to use a maternity waiting home. Experience of maternity waiting home (AOR = 3.337; 95% CI 2.038–5.463), direct subjective norm (AOR = 2.763; 95% CI 1.395–5.471), and direct perceived behavioral control (AOR = 23.147; 95% CI 2.341–4.231).

**Conclusion:**

In Ethiopia, the intention to use maternity waiting was low. There was an intentional variation in to use of maternity waiting homes across regions of Ethiopia. Improving behavioral perception through intervention programs such as antenatal education should have been strengthened.

**Supplementary Information:**

The online version contains supplementary material available at 10.1186/s40001-023-01248-7.

## Introduction

Every day, about 800 women worldwide die from pregnancy- or childbirth-related problems [1].

According to estimates, low- and middle-income nations account for about 99% of maternal and neonatal fatalities [2]. In sub-Saharan Africa, the maternal mortality rate in 2013 was 510 per 100,000 live births [3], primarily as a result of antepartum and postpartum hemorrhage, pregnancy-induced hypertension, botched abortion, and obstructed labor [4, 5]. Ethiopia continues to have one of the highest maternal death rates in the world. Maternal mortality was reported in 2016 to be 412 per 100,000 people in Ethiopia based on the demographic health survey [6].

When access to care is problematic, women with high-risk pregnancies should be admitted to a maternity waiting home at 36 weeks of gestation [7, 8]. Maternity Waiting Homes are short-term housing choices close to healthcare facilities where pregnant mothers wait to give birth, according to the World Health Organization [9]. It is a highly effective and affordable method for lowering maternal morbidity and mortality, and it provides a chance for residents in rural places to have access to trained birth attendants [10–12]. The use of maternal waiting home principles as a key tactic to reduce maternal and infant mortality was supported by data [13, 14].

An investigation showed that 27.3% of mothers in Zimbabwe and 10% of mothers in Kenya who gave birth in a hospital used a maternal waiting home [15]. The first maternity waiting home in Ethiopia was constructed in 1976 [16], and as of right now, the Amhara region has the highest maternity waiting home coverage (72%) followed by the Southern region (57%) and Oromia region (56%) and the lowest (8%) in Gambella region [17]. Although there have been maternal waiting homes in Ethiopia for more than 30 years, they are inaccessible to the majority of pregnant mothers [16, 18]. Overall, three delays contribute to maternal deaths: the decision to seek maternal health care, the journey to the facility, and the wait for care while there [19, 20]. A study found that maternal waiting at home improved access to a safe blood supply and the management of obstetric complications [18].

Maternal waiting homes have enhanced institutional delivery services and decreased maternal–child mortality. Evidence from Nigeria showed that maternal death ratio declined from 10 per 1000 to less than one per 1000 deliveries, and stillbirth rates decreased from 116 per 1000 to 20 per 1000 deliveries [21]. Institutional deliveries increased by 49% in Eritrea after the construction of a maternity waiting home [22]. Results in Tanzania also showed that the introduction of maternal waiting homes increased the number of deliveries in medical facilities and with trained birth attendants [12]. Maternal mortality rates among mothers who used maternity waiting homes were 89.9 per 100,000 live births lower than those of mothers who did not (1333.1 per 100,000 live births), and their rates of stillbirth were 17.6 per 1000 live births lower than those of mothers who did not (191 per 1000 live births), according to research from Ethiopia [23].

Even though maternal waiting homes help to improve maternity care, it may be challenging to receive these services due to logistical, financial, and regional limitations [7].

The expenditures of maternal waiting homes have been supported by community funding for several healthcare providers [23]. Due to limited accessibility, financial stress, a lack of transportation, and women's inability to select maternal waiting homes on their own, institutional deliveries are more common in low-resource countries [22].

There are no data at the national level, although many primary studies have supported Ethiopia's goal to employ maternity waiting homes. Therefore, this systematic review and meta-analysis study aimed to determine the pooled prevalence of maternity waiting for the home intention in Ethiopia and its determinants. Based on the study's results, clinicians and other stakeholders will be able to close gaps in intention to use maternity waiting homes and operational plans, giving them the essential information they need to give every childbearing woman.

## Methods

### Reporting

This systematic review and meta-analysis study was conducted to determine the intention to use maternity waiting homes and its predictors in Ethiopia using the standard PRISMA checklist guideline [24] (Additional file 1). The review protocol was published to PROSPERO, an international prospective register of systematic reviews, and assigned the identification number CRD42022354814.

#### Search strategy

International online databases (Pub Med, Science Direct, Scopus, and Google Scholar) were utilized to conduct a literature search on the prevalence of intention to use a maternal waiting home and its predictors in Ethiopia. We also retrieved gray literature from Addis Ababa University and Bahirdar University. Boolean operators “AND” and “OR” were used to construct the search string. The following core search terms and phrases with Boolean operators were used to search related articles: (“Maternal waiting home” OR “Maternity waiting area” OR “Maternity waiting facility” OR “Maternity waiting shelter”)) AND predictors) OR (“determinants” OR “associated factors”) AND Pregnant women) AND Ethiopia. The search was conducted using the following keywords and search terms “Intention”, “Willingness”, “Maternity waiting home”, “Utilization”, “Use”, “Maternal waiting home”, “Maternity waiting area”, “Maternity waiting shelter”, “Predictors”, “Determinants”, “associated factors”, “Pregnant women”, and “Ethiopia”. The search string in PubMed was: (((((((((Intention [tw] OR Willingness)) OR “Intention” [MeSH Terms]) AND Maternity waiting home [tw]) OR (“Maternity waiting home” [MeSH Terms] OR “maternal waiting home” [MeSH Terms] OR “Maternity waiting area” [MeSH Terms] OR “Maternity waiting facility” [MeSH Terms] OR “maternal waiting shelter” [MeSH Terms])) AND Predictor [tw]) OR (“Predictor” [MeSH Terms] OR “Determinant” [MeSH Terms] OR “Associated factor” [MeSH Terms])) AND Pregnant women [tw]) OR “Pregnant women” [MeSH Terms]) AND Ethiopia. Search terms were based on PICO principles to retrieve relevant articles through the databases mentioned above. The search period was from May 1/2022 to June 10/2022.

#### PECO guide

##### Population

Pregnant women.

##### Exposure

All pregnant women admitted to health facilities for delivery.

##### Comparison

Home birth.

##### Outcome

Intention to use maternal waiting home.

### Outcome measurement

Intention to use maternity waiting home: Intention to use maternity waiting home was measured using three questions: (1) I plan to use maternity waiting homes for the last remaining 2–4 weeks of my current pregnancy; (2) I will make my effort to use maternity waiting homes for my current pregnancy; (3) I intend to use maternity waiting homes for my current pregnancy. Each question contains five points Likert scales (1 = strongly disagree, 2 = disagree, 3 = neutral, 4 = agree, 5 = strongly agree). As a result, the total score was 3–15, and women who scored ≥ 60% were considered as women who intended to use maternal waiting homes [25].

Subjective norm: An individual's perception about using MWH, which is influenced by the judgment of significant others. it was measured by five-by-five Likert scale questions: (1) The majority of the people who care about me believe that I should use a maternity waiting home; (2) My decision to use a maternity waiting home or not is up to me; (3) The majority of the women in my village or neighborhood use MWHs; (4) It is expected of me to use an MWH; and (5) The majority of the people whose opinions I value would support my use of an MWH. There are five-point Likert scales for each question: 1 = strongly disagree, 2 = for disagree, 3 = for neutral, 4 = for agree, and 5 = for strongly agree. The total score ranged from 5 to 25. Women who scored between ≥ 60% were categorized to have positive subjective norms [25, 26].

Indirect subjective norm: It was measured by summing the product of normative belief with its corresponding motivation to comply.

Perceived behavioral control: It is the measure of an individual's belief concerning how easy or difficult it of using a maternity waiting home. There were three questions used to measure perceived behavioral control: (1) In our community, it is quite simple for me to use MWH; (2) if I choose to, I am certain that I can use MWH in the final 2–4 weeks of my pregnancy; and (3) Using MWHs is feasible in our system. Likert scales with five possible responses are included in each question (1 = strongly disagree, 2 = disagree, 3 = neutral, 4 = agree, and 5 = strongly agree). The total score ranged from 3 to 15 and women who had a score ≥ 60 were considered as positive perceived behavioral control [25, 26].

Indirect perceived behavioral control: It was measured by summing the product of control belief with its corresponding power.

### Inclusion and exclusion criteria

The papers that were included in this meta-analysis were those that were conducted in Ethiopia, were published in English, and had full texts that could be searched. Studies that included data on the intention to use maternity waiting homes were also reported. Qualitative studies, studies from other developed countries, research from duplicated sources, and articles missing the complete text were all omitted from this systematic review and meta-analysis. The eligibility of the included articles in this study was determined using the COCOPOP (Condition, Context, and Population) paradigm. Pregnant women made up the study population (POP), intending to use maternity waiting home serving as the condition (CO), and only studies carried out in Ethiopia serving as the context (CO).

### Quality assessment

After collecting the findings from all databases, the articles were exported to Microsoft Excel (2016) spreadsheet. Two authors (NA and KD) independently appraised the standard of the studies using the Joanna Briggs Institute (JBI) standardized quality appraisal checklist [27]. The disagreement raised during the quality assessment was resolved through a discussion led by the third author (MW). Finally, the argument was solved and reached an agreement. The critical analysis checklist has eight parameters with yes, no, unclear, and not applicable options. The parameters involve the following questions:

1. Where were the criteria for inclusion in the sample clearly defined?
2. Were the study subjects and, therefore, the setting described in detail?
3. Was the exposure measured result validly and reliably?
4. Were the main objective and standard criteria used to measure the event?
5. Were confounding factors identified?
6. Were strategies to affect confounding factors stated?
7. Were the results measured indeed and dependably? And (8) Was the statistical analysis suitable? Studies were considered low risk when they scored 50% and above on the quality assessment indicators, as reported in a supplementary file (Additional file 2).

### Risk of bias assessment

Two authors (NAG and KA) independently assessed included studies for risk of bias through the bias assessment tool developed by Hoy et al. [28], consisting of ten items that assess four domains of bias and internal and external validity. Any disagreement raised during the risk of bias assessment was resolved through a discussion led by the third author (YA). Finally, the argument was solved and reached an agreement. The first four items (items 1–4) evaluate the presence of selection bias, non-response bias, and external validity. The other six items (items 5–10) assess the presence of measuring bias, analysis-related bias, and internal validity. Therefore, studies that received ‘yes’ for eight or more of the ten questions were classified as ‘low risk of bias.’ If studies that received ‘yes’ for six to seven of the ten questions were classified as ‘moderate risk’ whereas studies that received ‘yes’ for five or fewer of the ten questions were classified as ‘high risk’ as reported in a supplementary file (Additional file 3).

### Data extraction

Microsoft Excel spreadsheet (2016) and STATA version 14 software were utilized for data extraction and analysis, respectively. Two authors (NAG and YA) independently extracted all relevant data using a standardized Joanna Briggs Institute data extraction format. The disagreement raised during data extraction was resolved through a discussion led by the third author (KA). Finally, the argument was solved and reached an agreement. The Joanna Briggs Institute standardized data extraction format was used to extract relevant data. The data automation tool was not used due to this study's absence of the paper form (manual data). The name of the first author, year of publication, study year, study region, study setting, study design, the prevalence of intention to use maternity waiting home, sample size, and quality of each paper was extracted.

#### Data analysis

After extracting all relevant findings in a Microsoft Excel spreadsheet, the data were exported to STATA software version 14 for analysis. The pooled prevalence of intention to use maternity waiting home was computed using a 95% confidence interval. Publication bias was checked by funnel plot and more objectively through Begg and Egger's regression tests, with *p* < 0.05 indicating potential publication bias [29]. The presence of between-study heterogeneity was checked by using the Cochrane Q statistic. This heterogeneity between studies was quantified using *I*^2^, in which a value of 0, 25, 50, and 75% represented no, low, medium, and high heterogeneity, respectively [30]. A forest plot was used to visually assess the presence of heterogeneity, which presented at a high-level random-effect model was used for analysis to estimate the pooled estimate of intention to use. Subgroup analysis was done by the study sub-region. A sensitivity analysis was executed to see the effect of a single study on the overall prevalence of the meta-analysis estimate. The findings of the study were presented in the form of text, tables, and figures.

## Results

### Search findings and study characteristics

**Fig. 1:**
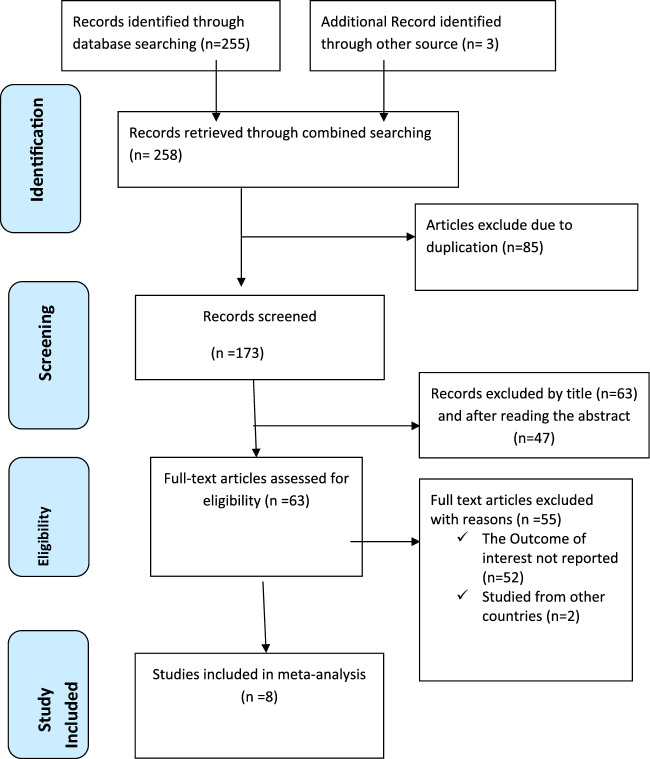
PRISMA diagram showing studies used for Systematic Review and Meta-analysis of the prevalence of episiotomy practice in Ethiopia

Through online search engines including PubMed, Scopus, Google Scholar, Science Direct, and online research repository home, 258 articles were found using a search strategy about the intention to use in Ethiopia. There were 173 articles left after duplicates were removed. The remaining studies' full titles and abstracts were then reviewed, and 110 studies were excluded. So, after 63 full-text studies were evaluated for eligibility, 55 articles were rejected for various reasons from consideration. Finally, this systematic review and meta-analysis study's criteria included 8 articles [26, 31–37] with 4111 study participants (Fig. 1).

All included studies employed by cross-sectional study design. Two of these were cross-sectional studies conducted at institutions, while the remaining six studies were community-based. Five studies were conducted in Southern Nations Nationalities and Peoples region [31–33, 35, 36], two in the Amhara region [26, 37], and one study in Oromia [34]. The sample sizes ranged from 322 to 829. The prevalence of intention to use maternity waiting homes ranged from 42.6% to 74.3%. All studies were assessed by using Joanna Briggs Institute (JBI) quality appraisal checklist and yielded low risk (Table 1).

**Table 1:** Characteristics of the included studies in the systematic review and meta-analysis for the prevalence of intention to use maternity waiting homes in Ethiopi**a**

Author year/Ref	Study area	Study region	Study setting	Study design	Sample size	Prevalence	Response	Quality
Gezimu et al./2021 [32]	Gamo sofa	SNNPR	Community	Cross-sectional	605	48.8	97.4	Low-risk
Worke yismaw/un-pub [31]	Mettu	SNNPR	Community	Cross-sectional	490	48.8	97.8	Low-risk
Nigussie et al./2020 [33]	Bench Maji	SNNPR	Community	Cross-sectional	829	42.6	98	Low-risk
Getinet Bayih Endalew/2016 [34]	Jimma	Oromia	Institutional	Cross-sectional	382	NA	98.7	Low-risk
Teshale Dojamo/un-pub [36]	Badwachaw	SNNPR	Community	Cross-sectional	556	44.6	99.1	Low-risk
Yohanis Terefe/un-pub [38]	Senan district	Amhara	Community	Cross-sectional	322	61.1	99	Low-risk
Endayehu et al./2020 [26]	Bellesa district	Amhara	Community	Cross-sectional	499	65.3	95	Low-risk
Vermiden et al./2018 [36]	Butajira	SNNPR	Institutional	Cross-sectional	428	55.1	100	Low-risk

### Meta-analysis

#### Prevalence of incomplete immunization among children in Ethiopia

**Fig. 2:**
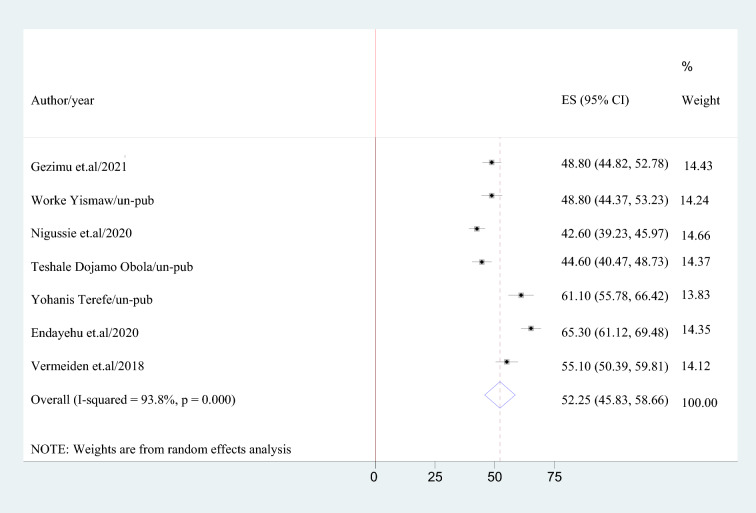
Pooled prevalence of intention to use maternity waiting home among women in Ethiopia

The total estimate of intention to use a maternity waiting home was calculated using a DerSimonian and Laird random-effects model. As a result, using a random-effects model, the national pooled prevalence of intention to use a maternity waiting home among childbearing women was 52.25% (95% CI 45.88–58.66), with a heterogeneity index (*I*^2^) of 93.8% (*p* = 0.001) (Fig. 2).

#### Subgroup analysis

**Fig. 3:**
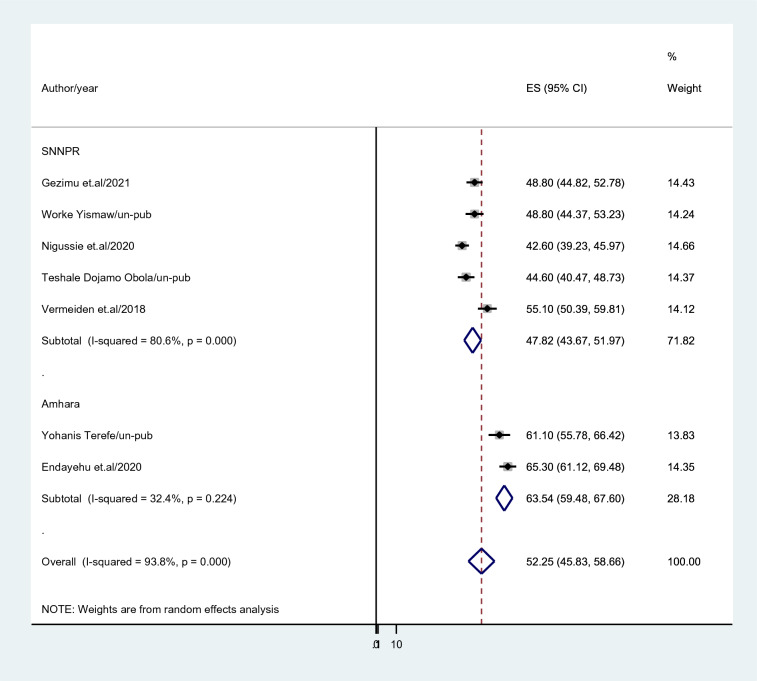
Subgroup analysis based region where study done

**Fig. 4:**
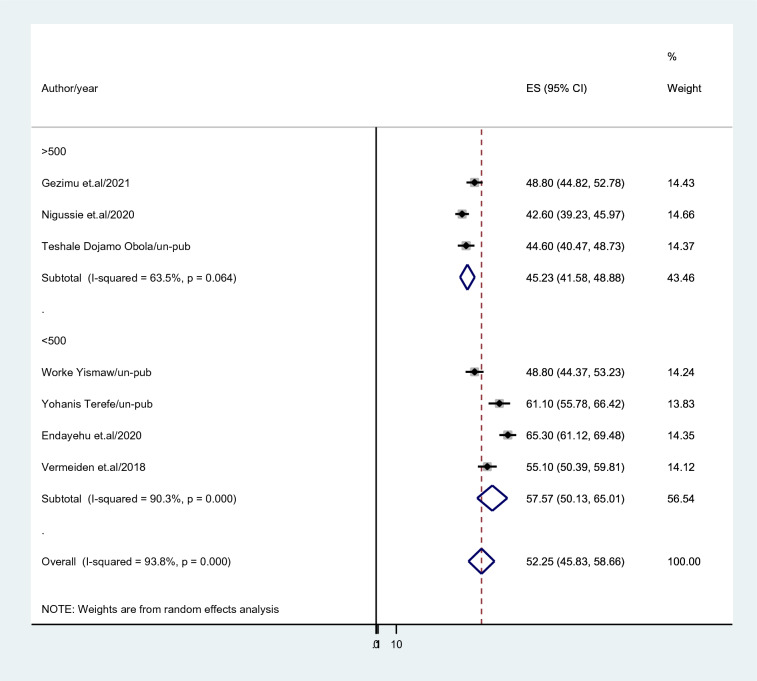
Subgroup analysis based on category of sample size

**Fig. 5:**
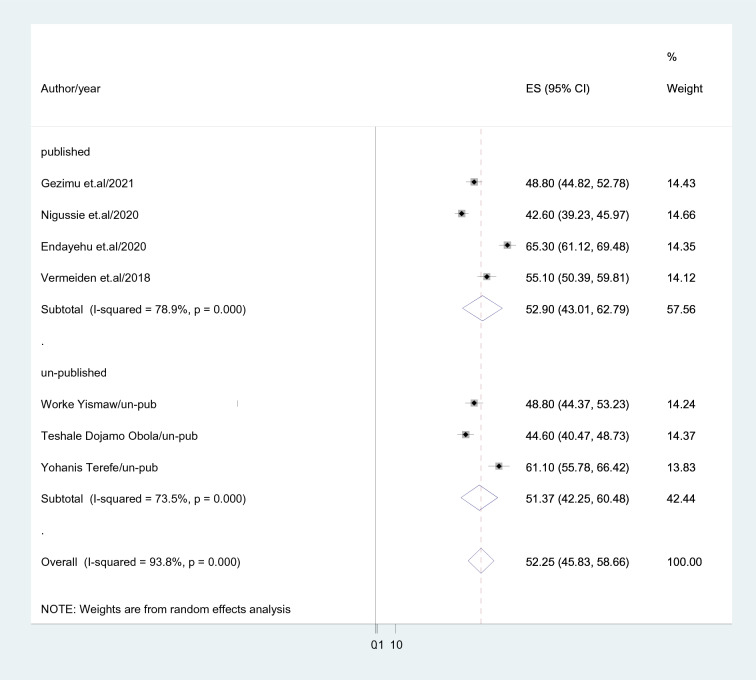
Subgroup analysis based on publication status of the included studies (published versus un-published)

Subgroup analysis based on region, publication, and sample size was carried out because this meta-analysis revealed a notable heterogeneity. Based on this, the Amhara region had a higher intention to utilize maternity waiting for home prevalence among childbearing women in Ethiopia (63.54%; 95% CI 59.48–67.60), *I*^2^ = 32.4%) than Southern Nations Nationalities and peoples region (47.82%; 95% CI 43.67–51.97), *I*^2^ = 80.6%) (Fig. 3). In studies with sample sizes less than 500, the prevalence of intention to use a maternity waiting home was reported as (57.57%; 95% CI 50.13–65.01); *I*^2^ = 90.3%, while for studies with sample sizes above 500, it was reported as (45.23%; 95% CI 41.58–48.88); *I*^2^ = 90.3% (Fig. 4). Intention to use a maternity waiting home was more prevalent in published research (52.9%; 95% CI 43.01–62.791); *I*^2^ = 78.9% than in unpublished studies (51.37%; 95% CI 42.25–60.48); *I*^2^ = 73.5% (Fig. 5).

#### Heterogeneity and publication bias

We calculated a subgroup analysis based on geography, sample size, and publication to correct the stated heterogeneity of this study (*I*^2^ = 93.8%). To pinpoint the cause of heterogeneity, Univar ate meta-regression was also carried out using the sample size and year as covariates. It was demonstrated that neither the sample size nor the year had any effect on the degree of research heterogeneity (Table 2).

**Table 2:** Meta-regression analysis of factors affecting between-study heterogeneit y

Heterogeneity source	Coefficients	Standard error	*p*-value
Sample size	4.293214	3.068427	0.211
Year	− 63.74237	68.4078	0.944

**Fig. 6:**
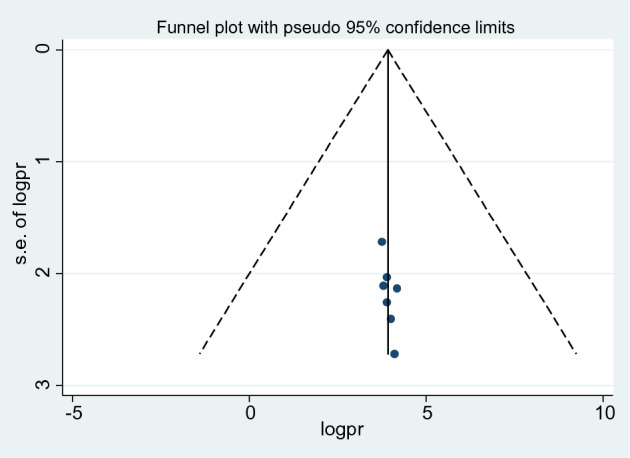
Forest plot symmetry displaying the absence of publication bias

The presence of publication bias was evaluated both visually using a funnel plot and objectively with Egger's test and Begg's test. By visual inspection, the funnel plot demonstrates a symmetrical distribution of studies (Fig. 6). Thus, the existence of publication bias was further evaluated using Egger's regression test (*p* = 0.010) and Begg's rank correlation test (*p* = 0.072), with no indication of publishing bias.

#### Leave-one-out-sensitivity analysis

By excluding one study at a time, a leave-one-out sensitivity analysis was used to determine the impact of each study on the overall prevalence of intending to use a maternity waiting home. Because of this, studies that were omitted individually did not reveal a marked change in the overall prevalence of intention to use a maternity waiting home (Table 3).

**Table 3:** The pooled prevalence of intention to use maternity waiting homes in Ethiopia when one study omitted from the analysis a step at a time

Study omitted	Estimate	95% CI
Gezimu et al./2021	52.842	45.213–60.470
Worke Yismaw/un-pub	52.832	45.328–60.336
Nigusie et al./2020	52.897	45.825–58.665
Getinet Bayih Endalew/2016	52.245	45.825–58.665
Teshale Dojamo Obola/un-pub	52.245	47.269–65.865
Yohanis Terefe/un-pub	50.822	44.070–57.574
Endayehu et al./2020	49.971	44.815–55.127
Vermeiden et al./2018	51.786	44.440–59.131

#### Factors associated with intention to use maternal waiting home in Ethiopia

In this meta-analysis, the intention to use a maternal waiting home was statically correlated with previous experience with maternal waiting homes, direct subjective norms, and direct perceived behavioral control. Positive attitude and the perception of indirect behavioral control, however, were not statistically associated to use a maternal waiting home. When there was variability between studies, the random-effect model was employed to determine effect sizes.

#### Favorable attitude

The results of this systematic review and meta-analysis showed no association between a positive attitude and the intention to use a maternal waiting home (AOR = 0.075; 95% CI 0.081–7.699). Given that *I*^2^ static showed a high level of heterogeneity (99.5%), the random-effect model was chosen for the analysis.

#### Indirect perceived behavioral control

In this study, there was no evidence of a significant relationship between intention to use maternal waiting home and indirect perceived behavioral control (AOR = 5.339; 95% CI 0.641–44.452). Considering that the value of *I*^2^ = 96.8 indicated the presence of heterogeneity, I used the random-effect model for the analysis.

#### Previous experience using maternal waiting home

**Fig. 7:**
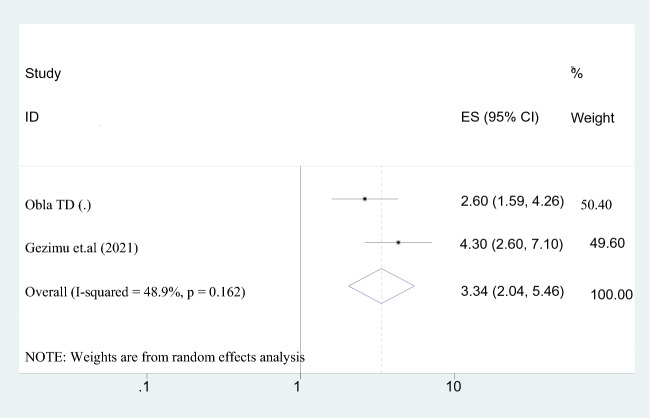
The association between past experience of maternity waiting home and intention to use maternal waiting home

According to this meta-analysis, women who had previously used a maternity waiting home were 3 times more likely to intend to utilize it than those who had not (AOR = 3.337; 95% CI 2.038–5.463). Assuming that the value of *I*^2^ was 48.9%, a random-effect model was suggested (Fig. 7).

#### Direct subjective norms

**Fig. 8:**
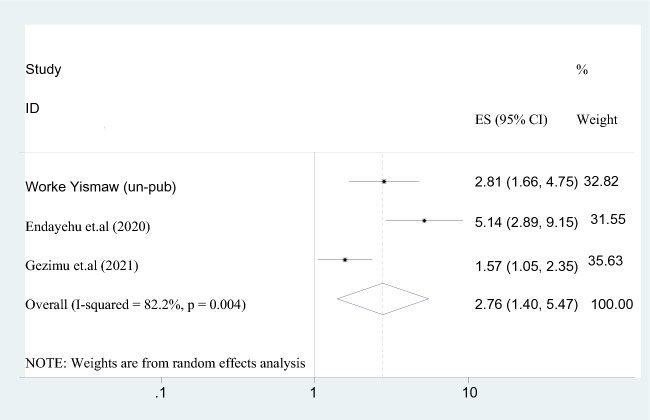
The association between direct subjective norm and intention to use maternity waiting home

When compared to indirect subjective norms, using maternity waiting homes was associated with a 2.8-fold higher likelihood (AOR = 2.763; 95% CI 1.395–5.471). The considerable heterogeneity (*I*^2^ = 67.1%) led to the adoption of a random-effect model (Fig. 8).

#### Direct perceived behavioral control

**Fig. 9:**
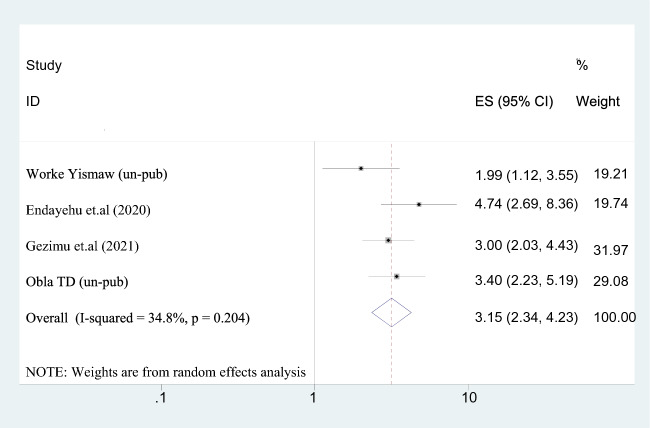
The association between direct perceived behavioral control and intention to use maternity waiting home

This study found that mothers who believed they had direct behavioral control over their actions were three times more likely to intend to use a maternity waiting home than those who believed they had none (AOR = 3.147; 95% CI 2.341–4.231). Because of the heterogeneity of the studies (*I*^2^ = 34.8.2%), the random-effect model was used (Fig. 9).

## Discussion

The majority of preventable maternal deaths are brought on by delayed or inaccessible maternal health services [38]. As a part of a comprehensive package of crucial obstetric services, maternal waiting homes are effective ways to bring women closer to obstetric care [39]. The Ethiopian Ministry of Health has established maternal waiting homes all around the country since 2014 [40]. The goal of this study was to determine maternal waiting home utilization intention among pregnant women in Ethiopia and its predictors. As a result, the pooled prevalence of intention to use maternity waiting homes in Ethiopia was 52.25% (95% CI 45.88–58.66). This result is consistent with a study conducted in Somaliland (58%) [41]. This may be because socioeconomic conditions in the two countries are comparable, which has a similar effect on health systems.

On the other hand, the result of the present study is higher than that of a study conducted in Kenya [42], which found that (45%) of the study participants intended to use a maternal waiting home. This discrepancy may be due to the length of time between studies and the differences in study participants. For instance, a study carried out in Kenya focused on participants from rural areas who might lack knowledge about and access to maternal waiting homes, whereas the current study included a mix of participants from both urban and rural areas.

The results of this study are less conclusive than those of studies conducted in Kenya (61,1%) [43], and Ghana (90%) [44] of the participants expressed a willingness to remain in maternity waiting homes. The setting of the study may be the reason, as this study mostly includes studies from the community, whereas other studies were conducted in a facility that allowed for an adequate understanding of maternity waiting homes. The variation in health policy and social structure between countries might be also mentioned as a possible justification.

Based on the region, sample size, and type of publication, subgroup analysis was performed.

Therefore, the subgroup analysis revealed that the Amhara region had a higher prevalence of intention to use maternity waiting homes (63.5%) than Southern Nation Nationalities and Peoples Region (47.8%). This discrepancy may have been caused by the Amhara region of Ethiopia having the highest coverage (72%) of maternity waiting homes [17].

Studies with sample sizes under 500 had a higher prevalence of intentions to use maternity waiting homes (57.6%) than studies with sample sizes over 500 (45.2%). This could be due to the number of studies and total sample size in each category For instance, there were three studies with sample sizes greater than 500 and five studies with sample sizes less than 500. However, the proportion of intention to use maternal waiting homes showed no noticeable difference between published research (52.9%) and unpublished studies (51.4%).

In this study, the intention to utilize maternal waiting homes was predicted by previous experience with maternal waiting homes, direct subjective norms, and direct perceived behavioral control. Women who had experience with maternity waiting homes were 3 times more likely intended to use maternity waiting homes than their counterparts. This finding is congruent with those studies conducted in Kenya [14], Zambia [15], and Somaliland [43]. A possible explanation for this similarity is that women who have previously used maternity waiting homes may know more about them, which improves their use of them.

The results of the current study show that the intention to use a maternity waiting home was significantly influenced by subjective norms. This result is consistent with research conducted in Somaliland [43]. This suggests that key referents including husbands, neighbors, fathers, in-laws, and health extension workers influence behavioral intention to use a maternity waiting home. Mothers require someone to take care of families who are left at home because using maternity waiting homes encourages women to abandon their homes and loved ones for days at a time. Another explanation could be that women in low- and middle-income countries have limited decision-making authority and that there are gender disparities in the decision-making process for maternal health services [45].

When compared to women who did not perceive any behavioral control, those with direct perceived behavioral control had higher odds of intending to use maternity waiting homes.

This result is in agreement with research done in Zambia [46]. The use of maternity waiting homes may be increased by empowering women to overcome barriers like transportation, food availability in maternity waiting homes, and water supply. Another explanation might be the women's understanding and perception of delivery complications could be monitored by maternal waiting for home use.

To handle a large variance that occurred in between-study heterogeneity, a random-effect model was used in this research. A leave-one-out sensitivity analysis was done, and the results reveal that no single study had a substantial effect on the overall prevalence of intention to use maternity waiting. Subgroup analysis was done based on region, sample size, and publication to see the presence of heterogeneity. The high heterogeneity might be due to differences in the sample populations, paper qualities, or socio-cultural, ethnic, and regional differences.

The study's findings are crucially significant since they address the maternity waiting for home gap and carefully provide evidence for the need for immediate adjustments in certain areas. Furthermore, these findings show policymakers in Ethiopia how to build maternity waiting homes and integration of services into their community health system. To improve maternity waiting home utilization, the workforce should encourage mothers to attend antenatal care appointments. It also demonstrates the significance of the experience of maternity waiting homes in reducing and correcting maternal and neonatal morbidity and mortality.

To the best of my knowledge, this is the first review that has pooled the national prevalence and identified comprehensive determinants. This study has some limitations. First, the absence of a similar previous study makes it very difficult to compare the findings of this study. Second, articles were restricted to only being prepared and published in the English language. Third, all of the included studies were cross-sectional, which might affect the outcome variable because of other confounding factors. Lastly, the use of women delivered at home as a comparison group has the potential risk of bias by overestimating the intention to use maternity waiting homes. This is because women directly admitted to these hospitals often attempt to give birth at home first, seeking medical care only when complications arise. This research has also some strengths. First, compressive electronic online international search engines were used. Second, our review incorporated grey literature as part of the primary studies. Third, the predictors for the intention to use maternity waiting homes were discovered. Lastly, the study protocol was registered in the prospective international register of systematic reviews (PROSPERO).

## Conclusion

In conclusion, the intention to use maternity waiting homes in Ethiopia was low. The pooled prevalence of intention to use maternal waiting homes differed based on region and sample size. Experience with maternity waiting homes, subjective norms, and direct perceived behavioral control were predictors of intention to use maternity waiting homes in Ethiopia. Improving behavioral perception through intervention programs such as antenatal education should have been strengthened.

### Supplementary Information

**Additional file 1. File S1-1. **Prisma checklist.

**Additional file 2. File S1-2. **Prisma checklist.

**Additional file 3. File S2. **Methodological quality assessment of included studies using Joanna Brigg's Institute quality appraisal criteria scale (JBI). The eight-item questions assessing inclusion criteria, study setting and participant, exposure measurement, objectives, confounder, statically analysis, outcome measurement, and dealing confounder were used.

**Additional file 4. File S3. **Risk of bias assessment for the included studies. The ten-item questions of which four items assess external and six items assess internal validity were used.

## Data Availability

All relevant data are within the Manuscript and its Supporting Information files.
