# Supplementary material for: Intention to use maternal waiting home and its predictors among pregnant women in Ethiopia: systematic review and meta-analysis
Source: Eur J Med Res. 2023 Aug 7;28:274. doi: 10.1186/s40001-023-01248-7 (PMC10405426; doi:10.1186/s40001-023-01248-7)
Supplement: Supplementary file 3 — Additional file 3. File S2. Methodological quality assessment of included studies using Joanna Brigg's Institute quality appraisal criteria scale (JBI). The eight-item questions assessing inclusion criteria, study setting and participant, exposure measurement, objectives, confounder, statically analysis, outcome measurement, and dealing confounder were used. [file 40001_2023_1248_MOESM3_ESM.docx]

Table2. Quality assessment for the included Studies

| Item | Clearly defined inclusion | Describe study setting and participant | Valid and reliable exposure measurement | Objective and standard criteria for measurement | Identified confounder | Strategies to deal with confounders | Valid and reliable outcome measurement | Appropriate statically analysis | No of ‘yes’s ‘ |
| --- | --- | --- | --- | --- | --- | --- | --- | --- | --- |
| Gezimu et.al | Yes | Yes | No | Yes | Yes | No | Yes | Yes | 6/8=75 |
| Worke Yismaw | Yes | Yes | Yes | Yes | No | No | Yes | Yes | 6/8=75 |
| Nigusie et.al | Yes | Yes | No | Yes | Yes | No | Yes | Yes | 6/8=75 |
| Getinet Bayih Endalew et.al | Yes | Yes | No | Yes | Yes | Yes | Yes | Yes | 7/8=87.5 |
| Teshale Dojamo | Yes | Yes | No | Yes | Yes | Yes | Yes | Yes | 7/8=87.5 |
| Endayehu et.al | Yes | Yes | Yes | Yes | Yes | No | Yes | Yes | 7/8=87.5 |
| Yohanis Terefe | Yes | Yes | No | Yes | Yes | Yes | Yes | Yes | 7/8=87.5 |
| Vermeiden et.al | Yes | Yes | Yes | Yes | No | No | Yes | Yes | 6/8=75 |
