# Supplementary material for: Intention to use maternal waiting home and its predictors among pregnant women in Ethiopia: systematic review and meta-analysis
Source: Eur J Med Res. 2023 Aug 7;28:274. doi: 10.1186/s40001-023-01248-7 (PMC10405426; doi:10.1186/s40001-023-01248-7)
Supplement: Supplementary file 4 — Additional file 4. File S3. Risk of bias assessment for the included studies. The ten-item questions of which four items assess external and six items assess internal validity were used. [file 40001_2023_1248_MOESM4_ESM.docx]

Table 2. Assessment of risk of bias for the included studies

| Item | External validity | | | | Internal validity | | | | | |  | |
| --- | --- | --- | --- | --- | --- | --- | --- | --- | --- | --- | --- | --- |
|  | Representativeness s of the target population | Representativeness s of the sampling frame | Radom sampling g or census | Minimal response e bias | Data were collected d directly | Acceptable e case definition used in the study | Valid and reliable measurement t | The same mode of data collection n for all study subject | Appropriate e length of prevalence period for parameter of interest | Appropriate numerators and denominator s of interest | No of yes | Summary of risk of bias |
| Gezimu et.al | Yes | Yes | No | Yes | Yes | No | Yes | Yes | Yes | Yes | 8 | Low-  risk |
| Worke Yismaw | Yes | Yes | Yes | Yes | No | No | Yes | Yes | Yes | Yes | 8 | Low risk |
| Nigusie et.al | Yes | Yes | No | Yes | Yes | No | Yes | Yes | Yes | Yes | 8 | Low- risk |
| Getinet Bayih  Endalew et.al | Yes | Yes | No | Yes | Yes | Yes | Yes | Yes | Yes | Yes | 9 | Low – risk |
| Tesha Dojamo | Yes | Yes | No | Yes | Yes | Yes | Yes | Yes | Yes | Yes | 9 | Low- risk |
| Endayehu et.al | Yes | Yes | Yes | Yes | Yes | No | Yes | Yes | Yes | Yes | 9 | Low- risk |
| Alfredo Douba | Yes | Yes | No | Yes | Yes | Yes | Yes | Yes | Yes | Yes | 9 | Low- risk |
| Yohanis Terefe | Yes | Yes | Yes | Yes | No | No | Yes | Yes | Yes | Yes | 8 | Low- risk |
| Vermedien et.al | Yes | Yes | No | Yes | Yes | Yes | Yes | No | Yes | Yes | 8 | Low- risk |
